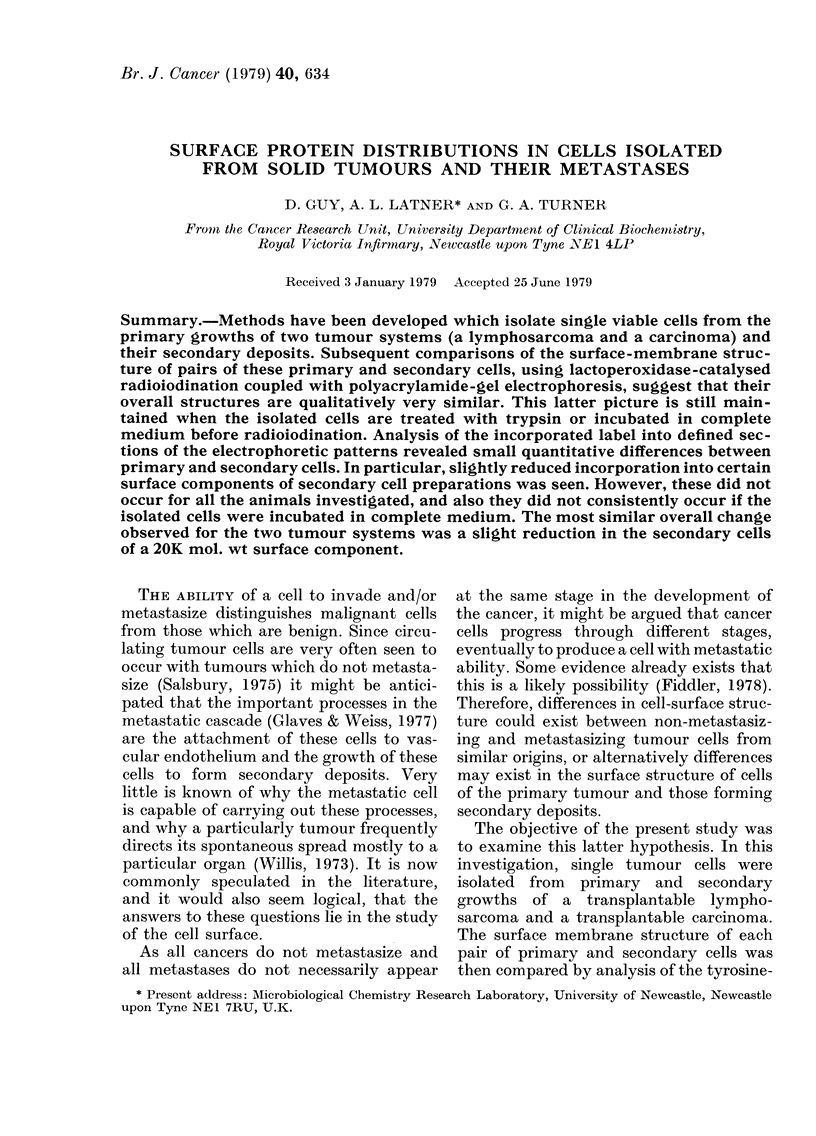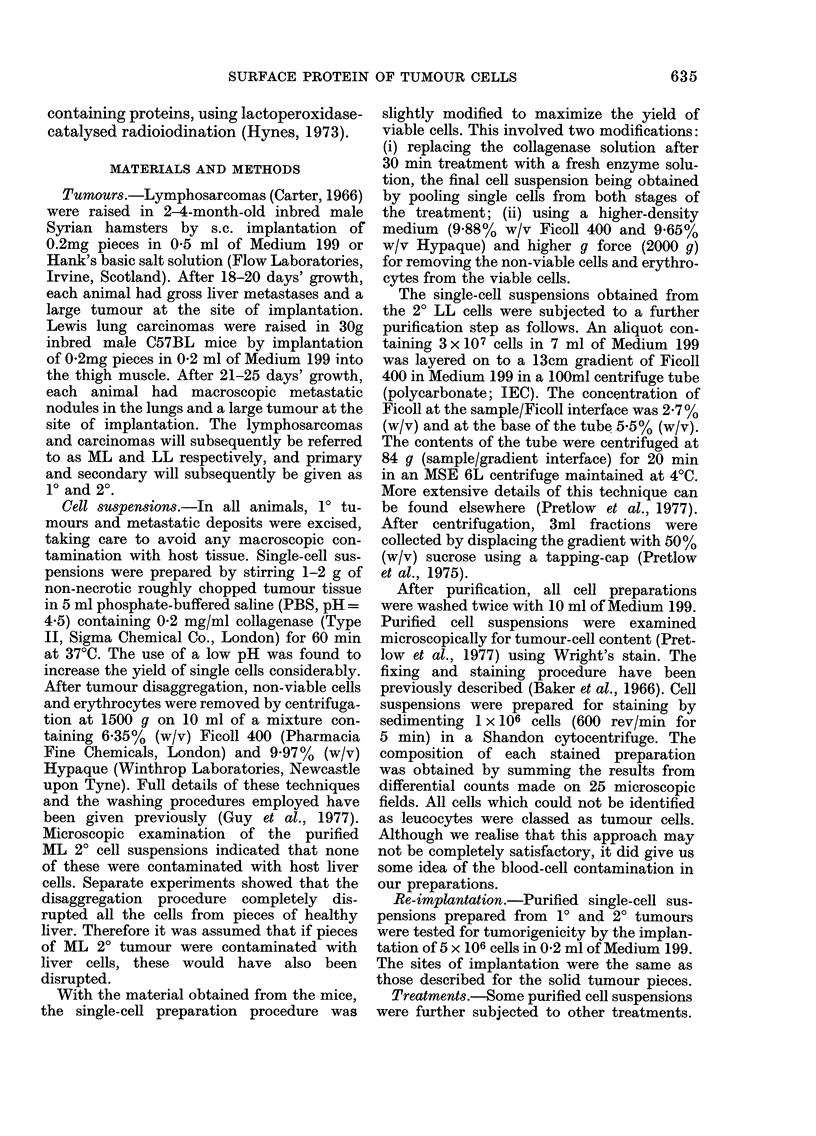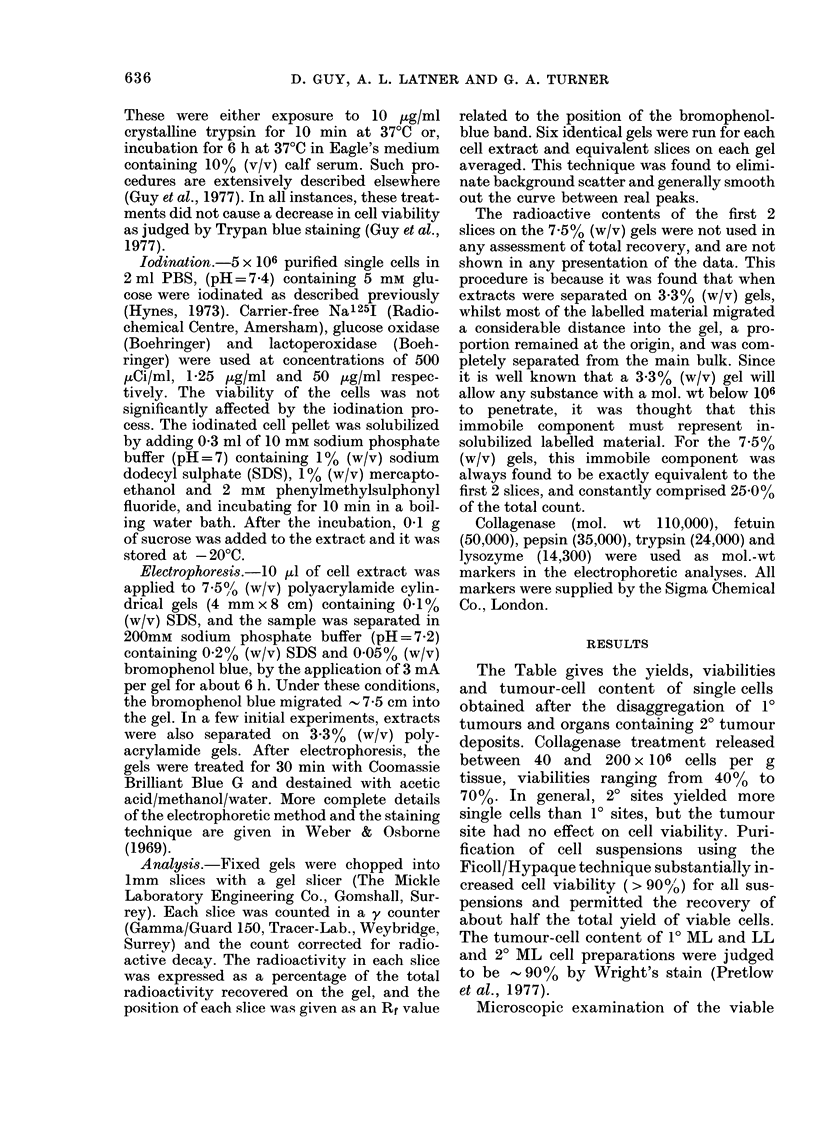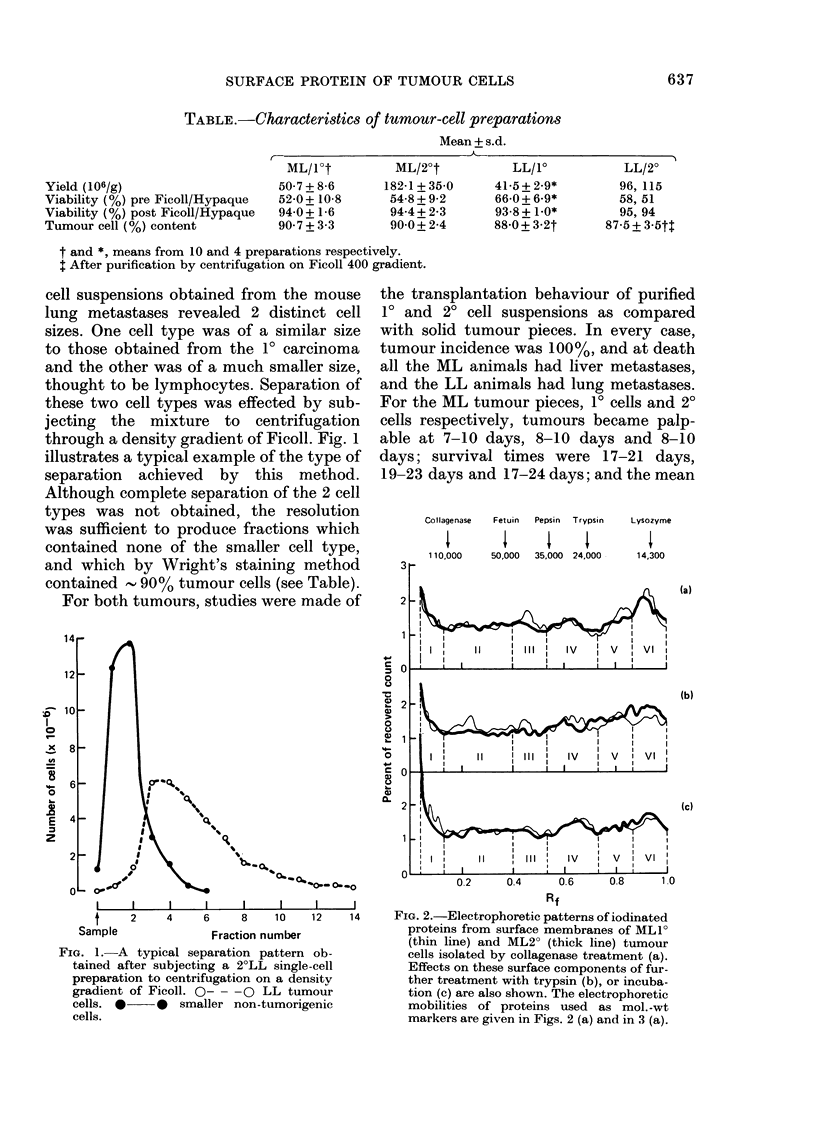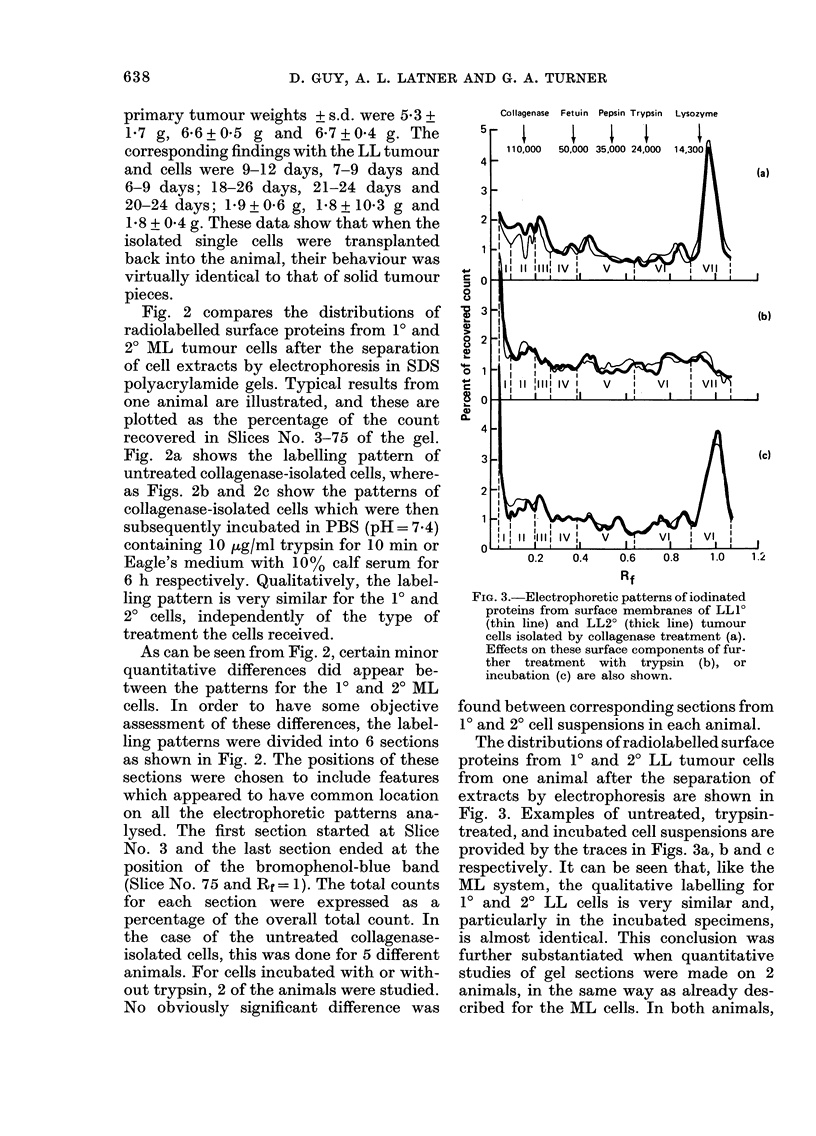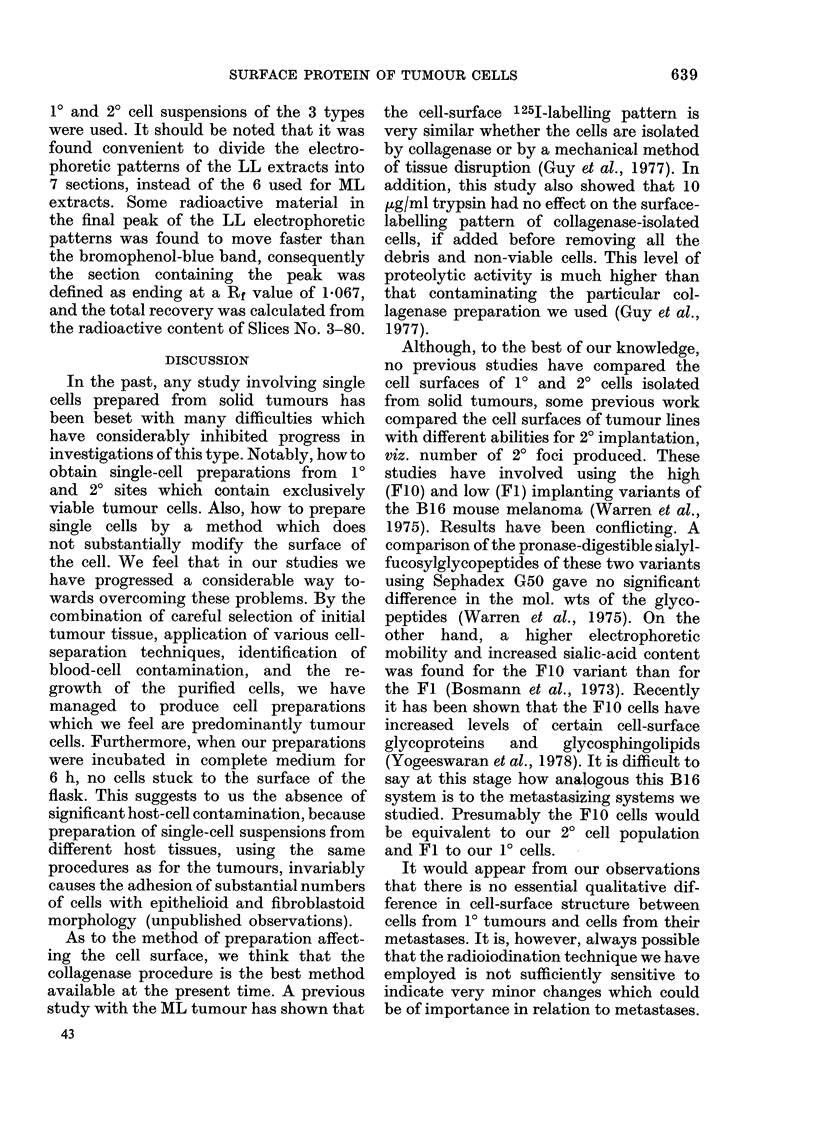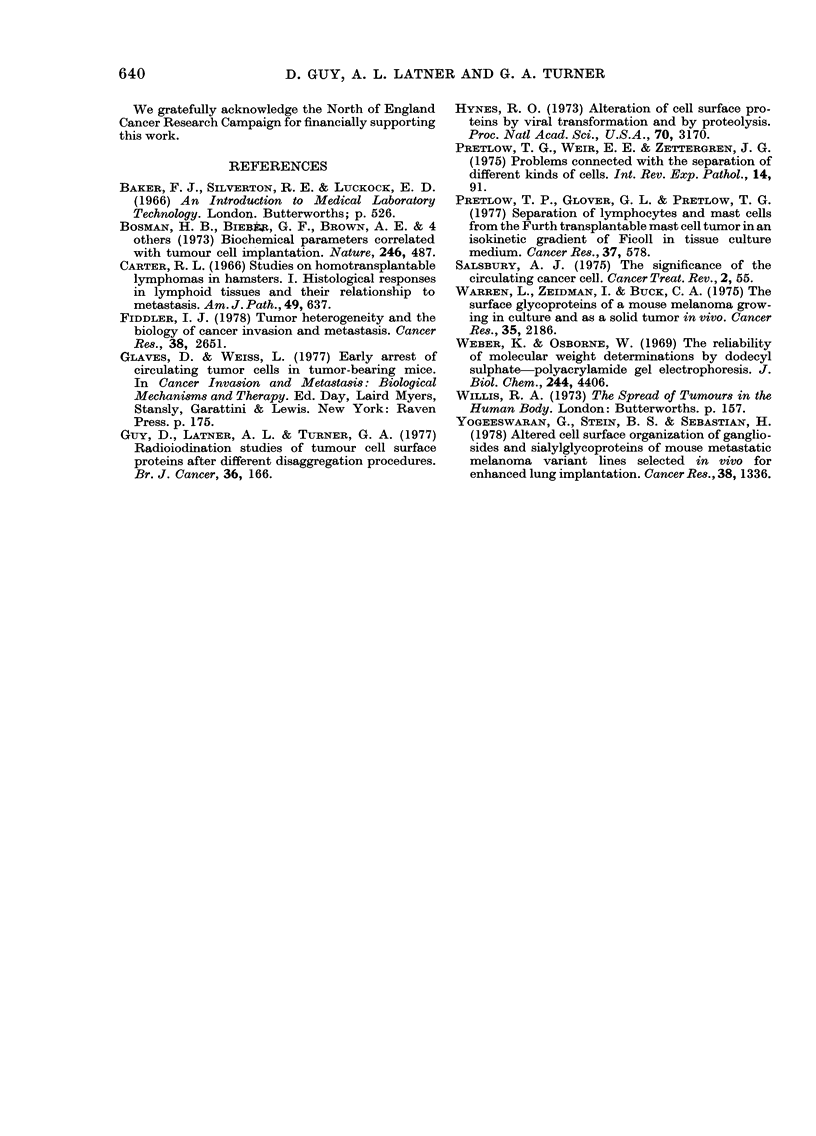# Surface protein distributions in cells isolated from solid tumours and their metastases.

**DOI:** 10.1038/bjc.1979.229

**Published:** 1979-10

**Authors:** D. Guy, A. L. Latner, G. A. Turner

## Abstract

Methods have been developed which isolate single viable cells from the primary growths of two tumour systems (a lymphosarcoma and a carcinoma) and their secondary deposits. Subsequent comparisons of the surface-membrane structure of pairs of these primary and secondary cells, using lactoperoxidase-catalysed radioiodination coupled with polyacrylamide-gel electrophoresis, suggest that their overall structures are qualitatively very similar. This latter picture is still maintained when the isolated cells are treated with trypsin or incubated in complete medium before radioiodination. Analysis of the incorporated label into defined sections of the electrophoretic patterns revealed small quantitative differences between primary and secondary cells. In particular, slightly reduced incorporation into certain surface components of secondary cell preparations was seen. However, these did not occur for all the animals investigated, and also they did not consistently occur if the isolated cells were incubated in complete medium. The most similar overall change observed for the two tumour systems was a slight reduction in the secondary cells of a 20K mol. wt surface component.


*Br. J. Cancer* (1979) **40**, 634

### SURFACE PROTEIN DISTRIBUTIONS IN CELLS ISOLATED FROM SOLID TUMOURS AND THEIR METASTASES

D. GUY, A. L. LATNER* AND G. A. TURNER

*From the Cancer Research Unit, University Department of Clinical Biochemistry, Royal Victoria Infirmary, Newcastle upon Tyne NE1 4LP*

Received 3 January 1979 Accepted 25 June 1979

**Summary.—Methods have been developed which isolate single viable cells from the primary growths of two tumour systems (a lymphosarcoma and a carcinoma) and their secondary deposits. Subsequent comparisons of the surface-membrane structure of pairs of these primary and secondary cells, using lactoperoxidase-catalysed radioiodination coupled with polyacrylamide-gel electrophoresis, suggest that their overall structures are qualitatively very similar. This latter picture is still maintained when the isolated cells are treated with trypsin or incubated in complete medium before radioiodination. Analysis of the incorporated label into defined sections of the electrophoretic patterns revealed small quantitative differences between primary and secondary cells. In particular, slightly reduced incorporation into certain surface components of secondary cell preparations was seen. However, these did not occur for all the animals investigated, and also they did not consistently occur if the isolated cells were incubated in complete medium. The most similar overall change observed for the two tumour systems was a slight reduction in the secondary cells of a 20K mol. wt surface component.**

THE ABILITY of a cell to invade and/or metastasize distinguishes malignant cells from those which are benign. Since circulating tumour cells are very often seen to occur with tumours which do not metastasize (Salsbury, 1975) it might be anticipated that the important processes in the metastatic cascade (Glaves & Weiss, 1977) are the attachment of these cells to vascular endothelium and the growth of these cells to form secondary deposits. Very little is known of why the metastatic cell is capable of carrying out these processes, and why a particularly tumour frequently directs its spontaneous spread mostly to a particular organ (Willis, 1973). It is now commonly speculated in the literature, and it would also seem logical, that the answers to these questions lie in the study of the cell surface.

As all cancers do not metastasize and all metastases do not necessarily appear at the same stage in the development of the cancer, it might be argued that cancer cells progress through different stages, eventually to produce a cell with metastatic ability. Some evidence already exists that this is a likely possibility (Fiddler, 1978). Therefore, differences in cell-surface structure could exist between non-metastasizing and metastasizing tumour cells from similar origins, or alternatively differences may exist in the surface structure of cells of the primary tumour and those forming secondary deposits.

The objective of the present study was to examine this latter hypothesis. In this investigation, single tumour cells were isolated from primary and secondary growths of a transplantable lymphosarcoma and a transplantable carcinoma. The surface membrane structure of each pair of primary and secondary cells was then compared by analysis of the tyrosine-

* Present address: Microbiological Chemistry Research Laboratory, University of Newcastle, Newcastle upon Tyne NE1 7RU, U.K.

containing proteins, using lactoperoxidase-catalysed radioiodination (Hynes, 1973).

### MATERIALS AND METHODS

*Tumours.*—Lymphosarcomas (Carter, 1966) were raised in 2–4-month-old inbred male Syrian hamsters by s.c. implantation of 0.2mg pieces in 0·5 ml of Medium 199 or Hank's basic salt solution (Flow Laboratories, Irvine, Scotland). After 18–20 days' growth, each animal had gross liver metastases and a large tumour at the site of implantation. Lewis lung carcinomas were raised in 30g inbred male C57BL mice by implantation of 0·2mg pieces in 0·2 ml of Medium 199 into the thigh muscle. After 21–25 days' growth, each animal had macroscopic metastatic nodules in the lungs and a large tumour at the site of implantation. The lymphosarcomas and carcinomas will subsequently be referred to as ML and LL respectively, and primary and secondary will subsequently be given as 1° and 2°.

*Cell suspensions.*—In all animals, 1° tumours and metastatic deposits were excised, taking care to avoid any macroscopic contamination with host tissue. Single-cell suspensions were prepared by stirring 1–2 g of non-necrotic roughly chopped tumour tissue in 5 ml phosphate-buffered saline (PBS, pH = 4·5) containing 0·2 mg/ml collagenase (Type II, Sigma Chemical Co., London) for 60 min at 37°C. The use of a low pH was found to increase the yield of single cells considerably. After tumour disaggregation, non-viable cells and erythrocytes were removed by centrifugation at 1500 *g* on 10 ml of a mixture containing 6·35% (w/v) Ficoll 400 (Pharmacia Fine Chemicals, London) and 9·97% (w/v) Hypaque (Winthrop Laboratories, Newcastle upon Tyne). Full details of these techniques and the washing procedures employed have been given previously (Guy *et al.*, 1977). Microscopic examination of the purified ML 2° cell suspensions indicated that none of these were contaminated with host liver cells. Separate experiments showed that the disaggregation procedure completely disrupted all the cells from pieces of healthy liver. Therefore it was assumed that if pieces of ML 2° tumour were contaminated with liver cells, these would have also been disrupted.

With the material obtained from the mice, the single-cell preparation procedure was slightly modified to maximize the yield of viable cells. This involved two modifications: (i) replacing the collagenase solution after 30 min treatment with a fresh enzyme solution, the final cell suspension being obtained by pooling single cells from both stages of the treatment; (ii) using a higher-density medium (9·88% w/v Ficoll 400 and 9·65% w/v Hypaque) and higher *g* force (2000 *g*) for removing the non-viable cells and erythrocytes from the viable cells.

The single-cell suspensions obtained from the 2° LL cells were subjected to a further purification step as follows. An aliquot containing $3 \times 10^7$ cells in 7 ml of Medium 199 was layered on to a 13cm gradient of Ficoll 400 in Medium 199 in a 100ml centrifuge tube (polycarbonate; IEC). The concentration of Ficoll at the sample/Ficoll interface was 2·7% (w/v) and at the base of the tube 5·5% (w/v). The contents of the tube were centrifuged at 84 *g* (sample/gradient interface) for 20 min in an MSE 6L centrifuge maintained at 4°C. More extensive details of this technique can be found elsewhere (Pretlow *et al.*, 1977). After centrifugation, 3ml fractions were collected by displacing the gradient with 50% (w/v) sucrose using a tapping-cap (Pretlow *et al.*, 1975).

After purification, all cell preparations were washed twice with 10 ml of Medium 199. Purified cell suspensions were examined microscopically for tumour-cell content (Pretlow *et al.*, 1977) using Wright's stain. The fixing and staining procedure have been previously described (Baker *et al.*, 1966). Cell suspensions were prepared for staining by sedimenting $1 \times 10^6$ cells (600 rev/min for 5 min) in a Shandon cytocentrifuge. The composition of each stained preparation was obtained by summing the results from differential counts made on 25 microscopic fields. All cells which could not be identified as leucocytes were classed as tumour cells. Although we realise that this approach may not be completely satisfactory, it did give us some idea of the blood-cell contamination in our preparations.

*Re-implantation.*—Purified single-cell suspensions prepared from 1° and 2° tumours were tested for tumorigenicity by the implantation of $5 \times 10^6$ cells in 0·2 ml of Medium 199. The sites of implantation were the same as those described for the solid tumour pieces.

*Treatments.*—Some purified cell suspensions were further subjected to other treatments.

These were either exposure to 10 μg/ml crystalline trypsin for 10 min at 37°C or, incubation for 6 h at 37°C in Eagle's medium containing 10% (v/v) calf serum. Such procedures are extensively described elsewhere (Guy *et al.*, 1977). In all instances, these treatments did not cause a decrease in cell viability as judged by Trypan blue staining (Guy *et al.*, 1977).

*Iodination.*—$5 \times 10^6$ purified single cells in 2 ml PBS, (pH = 7·4) containing 5 mM glucose were iodinated as described previously (Hynes, 1973). Carrier-free $Na^{125}I$ (Radiochemical Centre, Amersham), glucose oxidase (Boehringer) and lactoperoxidase (Boehringer) were used at concentrations of 500 μCi/ml, 1·25 μg/ml and 50 μg/ml respectively. The viability of the cells was not significantly affected by the iodination process. The iodinated cell pellet was solubilized by adding 0·3 ml of 10 mM sodium phosphate buffer (pH = 7) containing 1% (w/v) sodium dodecyl sulphate (SDS), 1% (w/v) mercaptoethanol and 2 mM phenylmethylsulphonyl fluoride, and incubating for 10 min in a boiling water bath. After the incubation, 0·1 g of sucrose was added to the extract and it was stored at −20°C.

*Electrophoresis.*—10 μl of cell extract was applied to 7·5% (w/v) polyacrylamide cylindrical gels (4 mm × 8 cm) containing 0·1% (w/v) SDS, and the sample was separated in 200mM sodium phosphate buffer (pH = 7·2) containing 0·2% (w/v) SDS and 0·05% (w/v) bromophenol blue, by the application of 3 mA per gel for about 6 h. Under these conditions, the bromophenol blue migrated ~7·5 cm into the gel. In a few initial experiments, extracts were also separated on 3·3% (w/v) polyacrylamide gels. After electrophoresis, the gels were treated for 30 min with Coomassie Brilliant Blue G and destained with acetic acid/methanol/water. More complete details of the electrophoretic method and the staining technique are given in Weber & Osborne (1969).

*Analysis.*—Fixed gels were chopped into 1mm slices with a gel slicer (The Mickle Laboratory Engineering Co., Gomshall, Surrey). Each slice was counted in a γ counter (Gamma/Guard 150, Tracer-Lab., Weybridge, Surrey) and the count corrected for radioactive decay. The radioactivity in each slice was expressed as a percentage of the total radioactivity recovered on the gel, and the position of each slice was given as an $R_f$ value related to the position of the bromophenol-blue band. Six identical gels were run for each cell extract and equivalent slices on each gel averaged. This technique was found to eliminate background scatter and generally smooth out the curve between real peaks.

The radioactive contents of the first 2 slices on the 7·5% (w/v) gels were not used in any assessment of total recovery, and are not shown in any presentation of the data. This procedure is because it was found that when extracts were separated on 3·3% (w/v) gels, whilst most of the labelled material migrated a considerable distance into the gel, a proportion remained at the origin, and was completely separated from the main bulk. Since it is well known that a 3·3% (w/v) gel will allow any substance with a mol. wt below $10^6$ to penetrate, it was thought that this immobile component must represent insolubilized labelled material. For the 7·5% (w/v) gels, this immobile component was always found to be exactly equivalent to the first 2 slices, and constantly comprised 25·0% of the total count.

Collagenase (mol. wt 110,000), fetuin (50,000), pepsin (35,000), trypsin (24,000) and lysozyme (14,300) were used as mol.-wt markers in the electrophoretic analyses. All markers were supplied by the Sigma Chemical Co., London.

### RESULTS

The Table gives the yields, viabilities and tumour-cell content of single cells obtained after the disaggregation of 1° tumours and organs containing 2° tumour deposits. Collagenase treatment released between 40 and $200 \times 10^6$ cells per g tissue, viabilities ranging from 40% to 70%. In general, 2° sites yielded more single cells than 1° sites, but the tumour site had no effect on cell viability. Purification of cell suspensions using the Ficoll/Hypaque technique substantially increased cell viability (> 90%) for all suspensions and permitted the recovery of about half the total yield of viable cells. The tumour-cell content of 1° ML and LL and 2° ML cell preparations were judged to be ~90% by Wright's stain (Pretlow *et al.*, 1977).

Microscopic examination of the viable

TABLE.—*Characteristics of tumour-cell preparations*

| | Mean ± s.d. | | | |
|---|---|---|---|---|
| | ML/1°† | ML/2°† | LL/1° | LL/2° |
| Yield ($10^6$/g) | 50·7 ± 8·6 | 182·1 ± 35·0 | 41·5 ± 2·9* | 96, 115 |
| Viability (%) pre Ficoll/Hypaque | 52·0 ± 10·8 | 54·8 ± 9·2 | 66·0 ± 6·9* | 58, 51 |
| Viability (%) post Ficoll/Hypaque | 94·0 ± 1·6 | 94·4 ± 2·3 | 93·8 ± 1·0* | 95, 94 |
| Tumour cell (%) content | 90·7 ± 3·3 | 90·0 ± 2·4 | 88·0 ± 3·2† | 87·5 ± 3·5†‡ |

† and *, means from 10 and 4 preparations respectively.
‡ After purification by centrifugation on Ficoll 400 gradient.

cell suspensions obtained from the mouse lung metastases revealed 2 distinct cell sizes. One cell type was of a similar size to those obtained from the 1° carcinoma and the other was of a much smaller size, thought to be lymphocytes. Separation of these two cell types was effected by subjecting the mixture to centrifugation through a density gradient of Ficoll. Fig. 1 illustrates a typical example of the type of separation achieved by this method. Although complete separation of the 2 cell types was not obtained, the resolution was sufficient to produce fractions which contained none of the smaller cell type, and which by Wright's staining method contained ~ 90% tumour cells (see Table).

For both tumours, studies were made of the transplantation behaviour of purified 1° and 2° cell suspensions as compared with solid tumour pieces. In every case, tumour incidence was 100%, and at death all the ML animals had liver metastases, and the LL animals had lung metastases. For the ML tumour pieces, 1° cells and 2° cells respectively, tumours became palpable at 7–10 days, 8–10 days and 8–10 days; survival times were 17–21 days, 19–23 days and 17–24 days; and the mean

FIG. 1.—A typical separation pattern obtained after subjecting a 2°LL single-cell preparation to centrifugation on a density gradient of Ficoll. ○– – –○ LL tumour cells. ●——● smaller non-tumorigenic cells.

FIG. 2.—Electrophoretic patterns of iodinated proteins from surface membranes of ML1° (thin line) and ML2° (thick line) tumour cells isolated by collagenase treatment (a). Effects on these surface components of further treatment with trypsin (b), or incubation (c) are also shown. The electrophoretic mobilities of proteins used as mol.-wt markers are given in Figs. 2 (a) and in 3 (a).

primary tumour weights ± s.d. were 5·3 ± 1·7 g, 6·6 ± 0·5 g and 6·7 ± 0·4 g. The corresponding findings with the LL tumour and cells were 9–12 days, 7–9 days and 6–9 days; 18–26 days, 21–24 days and 20–24 days; 1·9 ± 0·6 g, 1·8 ± 10·3 g and 1·8 ± 0·4 g. These data show that when the isolated single cells were transplanted back into the animal, their behaviour was virtually identical to that of solid tumour pieces.

Fig. 2 compares the distributions of radiolabelled surface proteins from 1° and 2° ML tumour cells after the separation of cell extracts by electrophoresis in SDS polyacrylamide gels. Typical results from one animal are illustrated, and these are plotted as the percentage of the count recovered in Slices No. 3–75 of the gel. Fig. 2a shows the labelling pattern of untreated collagenase-isolated cells, whereas Figs. 2b and 2c show the patterns of collagenase-isolated cells which were then subsequently incubated in PBS (pH = 7·4) containing 10 μg/ml trypsin for 10 min or Eagle's medium with 10% calf serum for 6 h respectively. Qualitatively, the labelling pattern is very similar for the 1° and 2° cells, independently of the type of treatment the cells received.

As can be seen from Fig. 2, certain minor quantitative differences did appear between the patterns for the 1° and 2° ML cells. In order to have some objective assessment of these differences, the labelling patterns were divided into 6 sections as shown in Fig. 2. The positions of these sections were chosen to include features which appeared to have common location on all the electrophoretic patterns analysed. The first section started at Slice No. 3 and the last section ended at the position of the bromophenol-blue band (Slice No. 75 and $R_f = 1$). The total counts for each section were expressed as a percentage of the overall total count. In the case of the untreated collagenase-isolated cells, this was done for 5 different animals. For cells incubated with or without trypsin, 2 of the animals were studied. No obviously significant difference was found between corresponding sections from 1° and 2° cell suspensions in each animal.

FIG. 3.—Electrophoretic patterns of iodinated proteins from surface membranes of LL1° (thin line) and LL2° (thick line) tumour cells isolated by collagenase treatment (a). Effects on these surface components of further treatment with trypsin (b), or incubation (c) are also shown.

The distributions of radiolabelled surface proteins from 1° and 2° LL tumour cells from one animal after the separation of extracts by electrophoresis are shown in Fig. 3. Examples of untreated, trypsin-treated, and incubated cell suspensions are provided by the traces in Figs. 3a, b and c respectively. It can be seen that, like the ML system, the qualitative labelling for 1° and 2° LL cells is very similar and, particularly in the incubated specimens, is almost identical. This conclusion was further substantiated when quantitative studies of gel sections were made on 2 animals, in the same way as already described for the ML cells. In both animals,

1° and 2° cell suspensions of the 3 types were used. It should be noted that it was found convenient to divide the electrophoretic patterns of the LL extracts into 7 sections, instead of the 6 used for ML extracts. Some radioactive material in the final peak of the LL electrophoretic patterns was found to move faster than the bromophenol-blue band, consequently the section containing the peak was defined as ending at a $R_f$ value of 1·067, and the total recovery was calculated from the radioactive content of Slices No. 3–80.

### DISCUSSION

In the past, any study involving single cells prepared from solid tumours has been beset with many difficulties which have considerably inhibited progress in investigations of this type. Notably, how to obtain single-cell preparations from 1° and 2° sites which contain exclusively viable tumour cells. Also, how to prepare single cells by a method which does not substantially modify the surface of the cell. We feel that in our studies we have progressed a considerable way towards overcoming these problems. By the combination of careful selection of initial tumour tissue, application of various cell-separation techniques, identification of blood-cell contamination, and the regrowth of the purified cells, we have managed to produce cell preparations which we feel are predominantly tumour cells. Furthermore, when our preparations were incubated in complete medium for 6 h, no cells stuck to the surface of the flask. This suggests to us the absence of significant host-cell contamination, because preparation of single-cell suspensions from different host tissues, using the same procedures as for the tumours, invariably causes the adhesion of substantial numbers of cells with epithelioid and fibroblastoid morphology (unpublished observations).

As to the method of preparation affecting the cell surface, we think that the collagenase procedure is the best method available at the present time. A previous study with the ML tumour has shown that the cell-surface $^{125}$I-labelling pattern is very similar whether the cells are isolated by collagenase or by a mechanical method of tissue disruption (Guy *et al.*, 1977). In addition, this study also showed that 10 μg/ml trypsin had no effect on the surface-labelling pattern of collagenase-isolated cells, if added before removing all the debris and non-viable cells. This level of proteolytic activity is much higher than that contaminating the particular collagenase preparation we used (Guy *et al.*, 1977).

Although, to the best of our knowledge, no previous studies have compared the cell surfaces of 1° and 2° cells isolated from solid tumours, some previous work compared the cell surfaces of tumour lines with different abilities for 2° implantation, *viz.* number of 2° foci produced. These studies have involved using the high (F10) and low (F1) implanting variants of the B16 mouse melanoma (Warren *et al.*, 1975). Results have been conflicting. A comparison of the pronase-digestible sialylfucosylglycopeptides of these two variants using Sephadex G50 gave no significant difference in the mol. wts of the glycopeptides (Warren *et al.*, 1975). On the other hand, a higher electrophoretic mobility and increased sialic-acid content was found for the F10 variant than for the F1 (Bosmann *et al.*, 1973). Recently it has been shown that the F10 cells have increased levels of certain cell-surface glycoproteins and glycosphingolipids (Yogeeswaran *et al.*, 1978). It is difficult to say at this stage how analogous this B16 system is to the metastasizing systems we studied. Presumably the F10 cells would be equivalent to our 2° cell population and F1 to our 1° cells.

It would appear from our observations that there is no essential qualitative difference in cell-surface structure between cells from 1° tumours and cells from their metastases. It is, however, always possible that the radioiodination technique we have employed is not sufficiently sensitive to indicate very minor changes which could be of importance in relation to metastases.

We gratefully acknowledge the North of England Cancer Research Campaign for financially supporting this work.

### REFERENCES

BAKER, F. J., SILVERTON, R. E. & LUCKOCK, E. D. (1966) *An Introduction to Medical Laboratory Technology*. London. Butterworths; p. 526.

BOSMAN, H. B., BIEBER, G. F., BROWN, A. E. & 4 others (1973) Biochemical parameters correlated with tumour cell implantation. *Nature*, **246**, 487.

CARTER, R. L. (1966) Studies on homotransplantable lymphomas in hamsters. I. Histological responses in lymphoid tissues and their relationship to metastasis. *Am. J. Path.*, **49**, 637.

FIDDLER, I. J. (1978) Tumor heterogeneity and the biology of cancer invasion and metastasis. *Cancer Res.*, **38**, 2651.

GLAVES, D. & WEISS, L. (1977) Early arrest of circulating tumor cells in tumor-bearing mice. In *Cancer Invasion and Metastasis: Biological Mechanisms and Therapy*. Ed. Day, Laird Myers, Stansly, Garattini & Lewis. New York: Raven Press. p. 175.

GUY, D., LATNER, A. L. & TURNER, G. A. (1977) Radioiodination studies of tumour cell surface proteins after different disaggregation procedures. *Br. J. Cancer*, **36**, 166.

HYNES, R. O. (1973) Alteration of cell surface proteins by viral transformation and by proteolysis. *Proc. Natl Acad. Sci., U.S.A.*, **70**, 3170.

PRETLOW, T. G., WEIR, E. E. & ZETTERGREN, J. G. (1975) Problems connected with the separation of different kinds of cells. *Int. Rev. Exp. Pathol.*, **14**, 91.

PRETLOW, T. P., GLOVER, G. L. & PRETLOW, T. G. (1977) Separation of lymphocytes and mast cells from the Furth transplantable mast cell tumor in an isokinetic gradient of Ficoll in tissue culture medium. *Cancer Res.*, **37**, 578.

SALSBURY, A. J. (1975) The significance of the circulating cancer cell. *Cancer Treat. Rev.*, **2**, 55.

WARREN, L., ZEIDMAN, I. & BUCK, C. A. (1975) The surface glycoproteins of a mouse melanoma growing in culture and as a solid tumor *in vivo*. *Cancer Res.*, **35**, 2186.

WEBER, K. & OSBORNE, W. (1969) The reliability of molecular weight determinations by dodecyl sulphate—polyacrylamide gel electrophoresis. *J. Biol. Chem.*, **244**, 4406.

WILLIS, R. A. (1973) *The Spread of Tumours in the Human Body*. London: Butterworths. p. 157.

YOGEESWARAN, G., STEIN, B. S. & SEBASTIAN, H. (1978) Altered cell surface organization of gangliosides and sialylglycoproteins of mouse metastatic melanoma variant lines selected *in vivo* for enhanced lung implantation. *Cancer Res.*, **38**, 1336.